# Impact of crumbled pelleted starter feed and alfalfa inclusion on feed intake, growth, and rumen microbiota in young lambs

**DOI:** 10.5713/ab.25.0007

**Published:** 2025-06-04

**Authors:** Qihao Gao, Guoxiu Wang, Zhanyu Chen, Jiale Jia, Haoyu Xu, Yunfei Xu, Zhen Liu, Liyun Liu, Baosheng Li, Chong Li

**Affiliations:** 1 College of Animal Science and Technology, Gansu Agricultural University, Lanzhou, China; 2 Gansu Runmu Bio-Engineering Co., Ltd., Yongchang, China

**Keywords:** Digestibility, Feed Technology, Pelleted Feeds, Rumen Fermentation, Rumen Microbes, Starter

## Abstract

**Objective:**

Pelleted feed has multiple advantages in animal production, but its hardness may limit the intake of young lambs with underdeveloped teeth and digestive systems, especially when the feed contains alfalfa and is produced with a high compression ratio in small diameters. This study aimed to evaluate the effects of removing alfalfa from starter feed or post-pelleting crumbling on lamb performance. We hypothesized that crumbling pelleted feed could reduce hardness, thereby increasing intake and enhancing performance.

**Methods:**

A total of 118 healthy, 7-day-old Hu lambs (4.02±0.94 kg) were allocated to three groups: (1) a pelleted starter with alfalfa (CON), (2) a non-alfalfa pelleted starter (NA), and (3) a crumbled starter with alfalfa (CA). Feed intake, growth performance, nutrient digestibility, and rumen microbial composition were measured.

**Results:**

The CA group demonstrated significantly reduced pellet hardness than the CON and NA groups (p<0.05), while NA group had higher starch gelatinization (p<0.05). CA notably increased feed intake, particularly after day 21 (p<0.05), and achieved the highest overall intake, body weight and average daily gain from days 7–49. The interaction between feed type and sex had significant impacton the digestibility of neutral detergent fiber (NDF) and acid detergent fiber (ADF). In male lambs, both NA and CA groups demonstrated significantly higher digestibility of NDF and ADF compared to the CON group (p<0.05). Rumen microbiota diversity was influenced by feed composition more than pellet form, with alfalfa inclusion affecting a greater number of microbial genera. Crumbling increased the abundance of *Methanobrevibacter* (p<0.05).

**Conclusion:**

These findings suggest that post-pelleting crumbling using a roller mill enhances feed intake and growth performance in young lambs, while feed composition plays a predominant role in shaping rumen microbial diversity.

## INTRODUCTION

Pelleted feed is widely used in the animal feed industry due to its ability to reduce ingredient segregation, minimize feed wastage, and improve both palatability and digestibility. In starter diets for lambs, its use has become increasingly common [1]. During pelleting, feed ingredients are conditioned under high temperatures for 15 to 20 seconds, which increases starch gelatinization and alters its molecular structure [2]. These changes can affect fermentation and digestion in the rumen [3]. Subsequently, the conditioned feed mixture is compressed through a die to form cylindrical pellets, which affects their hardness and density [4]. Research has shown that the physical form of feed plays an important role in starch digestion rates in ruminants, impacting their health and performance at various stages of growth [5]. However, studies on how pelleting affects lamb performance remain limited.

Alfalfa is commonly included in lamb starter feeds due to its nutritional benefits [6], but its inclusion presents notable processing challenges. As a high-quality forage, alfalfa provides essential nutrients including 18%–22% crude protein (CP) and 40%–45% neutral detergent fiber (NDF). Its appropriate fiber content can stimulate rumen development and epithelial maturation, and its bioactive compounds further contribute to the growth and development of lambs during the weaning transition [7]. These nutritional attributes make alfalfa an indispensable component in lamb starter diets, especially during the early stages of growth when the lambs’ rumens are still developing.

However, alfalfa’s high fiber content increases friction during pelleting, producing harder and denser pellets that may be less suitable for young lambs [8]. Young lambs’ underdeveloped teeth and digestive systems make pellet hardness a crucial factor in feed intake and digestion. During the processing of starter feeds, the use of smaller pellet diameters and higher compression ratios typically increases pellet hardness [9]. Therefore, it is necessary to improve the processing methods for lamb pellet feeds to reduce the negative impact of pellet hardness on lamb intake while preserving the developmental benefits of alfalfa.

To solve these problems, producing large pellets using low compression ratio ring dies followed by a post-pelleting crumbling process, may improve feed intake in young lambs. This method involves pressing cooled pellets through a roller crumbler and grading sieves to create lamb-appropriate small pellets, reducing fine particles. Although this approach is widely used in chick feed production [10], its application to lamb starter feed has not been studied. Some studies have highlighted the benefits of pelleted feeds on lamb feed intake, growth performance, and rumen fermentation [11]; however, using a post-pelleting crumbling process to reduce excessive pellet hardness may further enhance the effectiveness of pelleted feeds for young lambs.

This study aims to evaluate the effects of removing alfalfa from pelleted starter feeds or using post-pelleting crumbling on feed intake, growth performance, nutrient digestibility, rumen fermentation, and microbiota composition in lambs. We hypothesize that removing alfalfa or applying post-pelleting crushing can effectively reduce feed hardness, thereby improving lamb feed intake and growth performance. Regarding alfalfa’s capacity to stimulate rumen development, the crumbling process after pelleting is expected to offer additional benefits by improving feed palatability through texture modifying while maintaining alfalfa’s stimulatory effect on rumen development.

## MATERIALS AND METHODS

### Experimental treatments, feeding, and measurements of growth performance

A total of 131 Hu lambs born within a 3-day period were pre-assigned to three dietary treatment groups at birth and transferred with their mothers into designated pens (6 m×4 m) according to group allocation. After excluding weak lambs requiring intensive care and their siblings before 7 days of age to maintain maternal-group integrity, 118 healthy lambs (4.02± 0.94 kg body weight [BW]) remained for the formal trial, with final group sizes as follows: Control Group (CON, n = 38; 18 males and 20 females): Fed a standard pelleted starter feed containing alfalfa, with a pellet diameter of 4.5 mm. Non-Alfalfa Group (NA, n = 43; 20 males and 23 females): Fed a standard pelleted starter feed without alfalfa, also with a pellet diameter of 4.5 mm. Crumbled Alfalfa Group (CA, n = 37; 18 males and 19 females): Fed a starter feed with the same formulation as the CON group, but processed with a pellet mill using a 6 mm diameter die (selected because larger-diameter dies produce pellets with lower compression ratios and consequently reduced hardness, thereby optimizing feed hardness for lamb palatability), followed by crumbling with a roller mill (SSLG15×80 twin-roller crusher; Jiangsu Degao Machinery; 3–4 t/h capacity). The crumbled material was then passed through a vibrating sieve to remove undersized particles and fines, which were recycled back into the pelleting process. This sieving step ensured consistent particle size distribution while maintaining pellet durability. The final crumbled starter feed had a controlled particle size of 4.04±1.45 mm. The composition and nutritional content of the starter feeds are presented in Table 1. All formulations were consistent across the treatments and produced from the same batch of raw materials to minimize variability.

All lambs were allowed free nursing with their mothers after birth. At 7 days of age, they were transferred with their mothers into designated pens (6 m×4 m) based on their assigned experimental group. Each treatment group was randomly divided into three pens, for a total of nine pens. Pens were equipped with a supplementary feeding area for lambs (1 m×2 m) where starter feed was provided. This area was designed to restrict access by ewes, ensuring that only lambs could consume the starter feed. Starter feed was supplied daily at 8:00 a.m., and lambs had ad libitum access to nursing, water, and starter feed throughout the experimental period. Ewes were fed the same total mixed ration diet, administered twice daily, to ensure uniform maternal nutrition across all groups.

From 7 days of age, lamb BW, average daily gain (ADG), and starter feed intake were measured every 14 days. The evening before each BW measurement, all starter feed was removed from the troughs to ensure fasting, and lambs were weighed the following morning in a fasted state. Starter feed intake was calculated as the difference between the amount of feed offered and the feed remaining. Feed offered and refusals were recorded daily to ensure accurate measurements.

### Determination of physical and chemical properties of pellet feed

For each feed group, three samples of approximately 500 g were collected to evaluate hardness, density, and starch gelatinization. Twenty pellets of uniform length and intact appearance were selected using a quartering method. These pellets were compressed along their diameters until fracture using a hardness tester (YHKC-2A; Taizhou Galaxy Instruments). The mean pressure required for breakage was recorded as the hardness value for each group. A 100 g feed sample was placed into a graduated syringe, and the scale was recorded. A vacuum was applied to measure the inter-pellet void volume. The total pellet volume was then calculated, and density was derived using the formula: *ρ* = *m*/*V*, where *ρ* is the density (g/cm^3^), *m* is the mass of the sample (g), and *V* is the volume (cm^3^). Starch gelatinization was measured following the method described by Xiong [12], ensuring consistency with previously established procedures.

### Sample collection and measurement of nutrient digestion

Eighteen lambs (6 from each feed group, with 3 males and 3 females per group) were selected for fecal sample collection. Following weaning at 49 days of age and a 5-day transition period, fecal samples were collected twice daily over four consecutive days using rectal collection. The samples were pooled for each lamb. A portion of the pooled fecal samples was treated with 10% sulfuric acid for nitrogen fixation, followed by CP analysis. The remaining samples were sealed in plastic bags, dried at 65°C, and later used to determine dry matter (DM), NDF, acid detergent fiber (ADF), and acid-insoluble ash content. Acid-insoluble ash was employed as an internal marker to calculate the apparent digestibility of CP, DM, NDF, ADF, and organic matter (OM). The starter feed and fecal samples were analyzed for: DM: Determined by drying samples at 105°C. CP: Analyzed according to AOAC International standards [13]. Acid-insoluble ash: Determined using a previously described method [14]. NDF and ADF: Analyzed using a previously described method incorporating heat-stable alpha-amylase and sodium sulfate in the NDF procedure [15]. The apparent digestibility of DM, OM, CP, NDF, and ADF was calculated using the following equation:


(1) $$\begin{array}{l} \text{Nutrient apparent digestibility }(\%)=\\ 100\%-(\text{Acid-insoluble ash content in feces}/\\ \text{Acid-insoluble ash content in feed}\times \text{Nutrient content in}\\ \text{feed}/\text{Nutrient content in feces})\times 100\% \end{array}$$
[14]

### Collection of rumen fluid and measurement of volatile fatty acids

At 49 days of age, 50 mL of rumen fluid was collected from each lamb using a custom-designed oral rumen tube in the morning while the animals were fasting. The tube featured a polished copper tip specifically sized for lambs’ small rumen capacity, with a smooth, streamlined design to minimize tissue damage. Prior to each sampling procedure, the tube was thoroughly sterilized to prevent any potential infection. During sampling, lambs were properly restrained and the tube was inserted and withdrawn slowly and carefully to avoid any damage to the digestive tract. The collected rumen fluid was immediately transferred into cryovials and frozen at −20°C for subsequent analysis of fermentation parameters and microbial sequencing.

The concentrations of volatile fatty acids (VFAs) in the rumen fluid were determined using gas chromatography (GC) [16]. The rumen fluid samples were pretreated by filtration through a 0.45 μm disposable filter to remove particulates. The clear supernatant was then transferred to vials for GC analysis. VFA concentrations were quantified using a Shimadzu gas chromatograph (GC-2010Plus) with 2-ethylbutyric acid (2-EB) as the internal standard.

### Extraction of microbial DNA from rumen fluid and high-throughput sequencing

Total DNA was extracted from rumen fluid using the Omega E.Z.N.A. Stool DNA Kit (Omega Bio-Tek). The quality and concentration of the extracted DNA were assessed using a NanoDrop 2000 spectrophotometer (NanoDrop Technologies). DNA was diluted to a concentration of 50 ng/μL for downstream applications.

For microbial community analysis, the V3–V4 regions of the 16S rRNA gene were amplified using universal primers 341-F (5′-CCTAYGGGRBGCASCAG-3′) and 806-R (5′-GGACTACNNGGGTATCTAAT-3′). The PCR conditions were as follows: an initial denaturation at 98°C for 1 minute, followed by 30 cycles of denaturation at 98°C for 10 seconds, annealing at 50°C for 30 seconds, and elongation at 72°C for 30 seconds, with a final extension at 72°C for 5 minutes. Barcoded amplicons were pooled in equimolar amounts for Illumina paired-end library preparation. Sequencing was performed on an Illumina NovaSeq 6000 platform, producing 250 bp paired-end reads.

Paired-end reads were merged using FLASH (v1.2.11) to create raw tags. Tags were quality-filtered using the QIIME toolkit, resulting in high-quality sequences termed “Effective Tags.” These sequences were denoised with the DADA2 or deblur modules in QIIME2 (version 202202), generating amplicon sequence variants (ASVs). Taxonomic assignment was performed using the SILVA database within mothur, with a confidence threshold of 0.80.

Metrics including Chao1, Shannon, Simpson, ACE, and Observed Species indices were calculated to evaluate within-sample species diversity using QIIME2. Differences in microbial communities across samples were analyzed using Principal Coordinates Analysis (PCoA) and cluster analysis, performed in QIIME (version 1.8.0).

### Statistical analysis

The data were analyzed using SPSS (version 26.0; IBM). A two-way analysis of variance (ANOVA) within the general linear model framework was applied to assess the main effects of starter feed type and sex, as well as their interactions, on growth performance, nutrient digestion, and rumen fermentation indices. To ensure robust statistical analysis, Type III sum of squares was selected to account for the unbalanced experimental design. All ANOVA assumptions were verified, including homogeneity of variance and residual normality. Post hoc multiple comparisons were conducted using the least significant difference method. Statistical significance was defined as p<0.05, while values within the range of 0.05<p<0.1 were interpreted as indicating a trend toward significance.

## RESULTS

### Physical properties and starch gelatinization of starter feeds with different forms

As shown in Table 2, the hardness of the pelleted feed in the CA group was significantly lower compared to the CON and NA groups (p<0.05). The degree of starch gelatinization in the NA group was significantly higher than that in the CON and CA groups (p<0.05). However, no significant differences were observed in the density of the feeds among the three groups (p>0.05).

### Effects of different starter feeds on lamb growth performance

As presented in Table 3, the interaction between feed type and sex did not significantly affect growth performance (p>0.05). Sex significantly affected BW before 35 days and ADG before 21 days, with males showing higher values than females (p< 0.05). Feed type significantly influenced the ADG of lambs during the periods of 7–21 days, 35–49 days, and 7–49 days (p<0.05). Specifically: From 7–21 days, the ADG of the CA group was significantly lower than that of the CON and NA groups. From 35–49 days, the ADG of the CA group was significantly higher than that of the other two groups. Overall, the ADG of the CA group from 7–49 days was significantly higher than that of the NA group (p<0.05). At 49 days, the BW of the CA group tended to be higher than that of the CON and NA groups (p<0.1).

### Effects of different starter feeds on lamb starter feed intake

As presented in Table 4, feed intake was significantly greater in the CA group compared to the CON and NA groups across all stages (p<0.05), with a pronounced increase after 21 days, where the intake of the CA group substantially exceeded that of the other two groups (p<0.05).

### Effects of different starter feeds on nutrient digestibility in lambs

As shown in Table 5, the interaction between feed type and sex significantly influenced the digestibility of NDF and ADF. Subsequent simple effects analysis demonstrated that in male lambs, both NA and CA groups exhibited significantly greater NDF and ADF digestibility compared to CON (p<0.05); whereas no significant differences were detected among dietary treatments in female lambs (p>0.05). The digestibility of CP in the CA group showed a trend toward being higher compared to the CON and NA groups (p<0.1). Sex significantly affected CP digestibility, with male lambs exhibiting higher values than female lambs (p<0.05). However, sex did not significantly affect the digestibility of other nutrients (p>0.05). Feed type, sex, and their interaction did not significantly influence the digestibility of DM or OM (p>0.05).

### Effects of different starter feeds on rumen fermentation in lambs

As shown in Table 6, the interaction between feed type and sex did not significantly affect the concentration of any VFAs in lambs (p>0.05). While feed type showed no significant effect on total VFAs or several individual acids (acetic, isobutyric, butyric, isovaleric; p>0.05), it significantly affected the concentration of propionic acid (p<0.05) and showed a trend toward influencing valeric acid concentration (p<0.1). Specifically, the NA group exhibited lower concentrations of propionic and valeric acids compared to the CON group (p<0.05). Sex significantly influenced the concentrations of propionic acid and isobutyric acid, with male lambs displaying lower levels than female lambs (p<0.05).

### Effects of different starter feeds on rumen microbial communities in lambs

**Figure 1:**
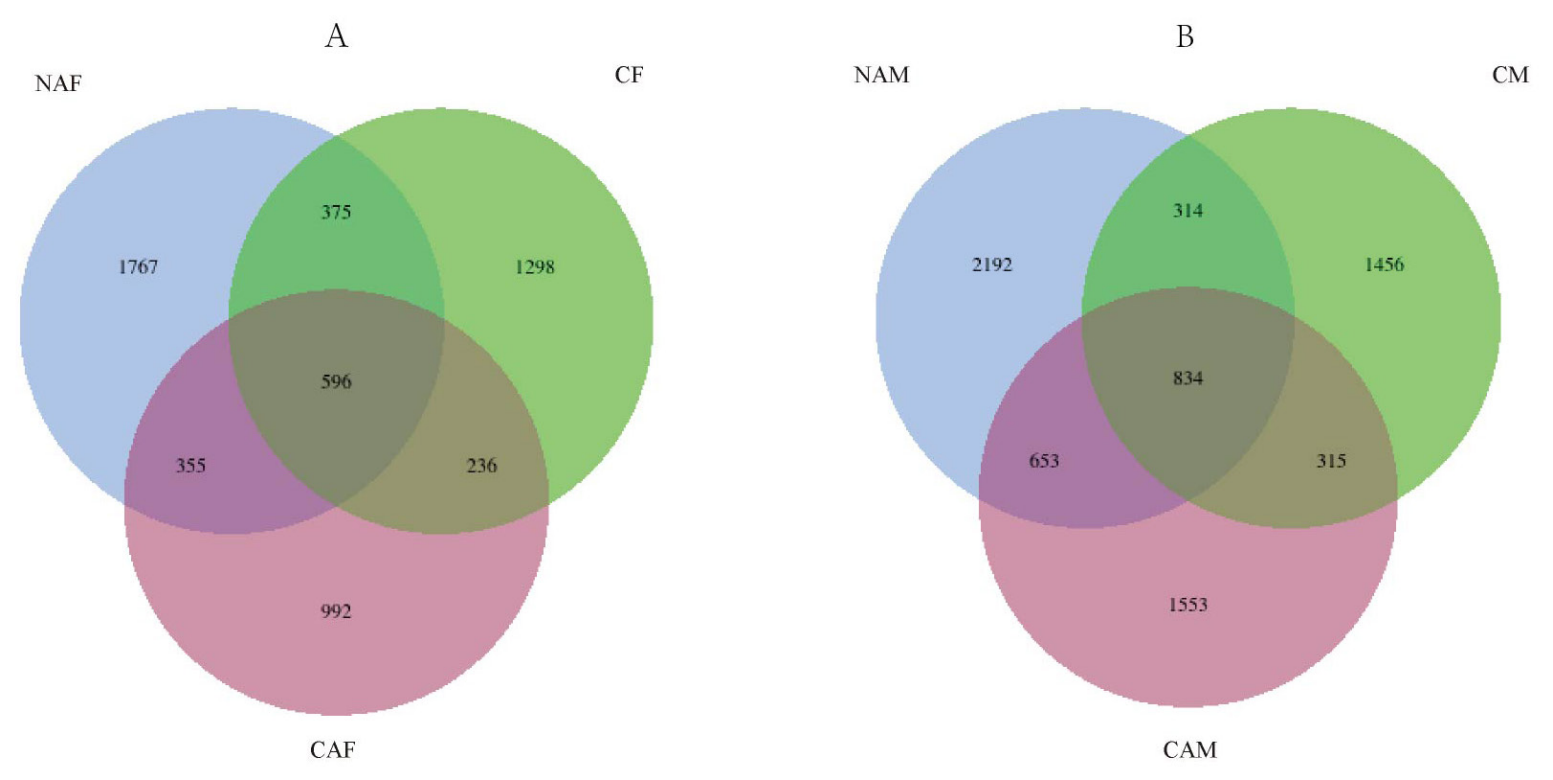
Relationship between different starter feeds and rumen microbial diversity in lambs. Differences in amplicon sequence variants (ASVs) were assessed among the groups. NAF, standard pelleted starter feed without alfalfa, female lambs; CF, standard pelleted starter feed containing alfalfa, female lambs; NAM, standard pelleted starter feed without alfalfa, male lambs; CM, standard pelleted starter feed containing alfalfa, male lambs; CAF, starter feed with the same formulation as the CON group, but crumbled using a roller mill after pelleting, female lambs; CAM, starter feed with the same formulation as the CON group, but crumbled using a roller mill after pelleting, male lambs; F, female; M, male.

*16S rDNA sequencing results of rumen microbes:* An average of 105,235 rRNA gene reads were obtained from the rumen fluid samples across all groups. Analysis using QIIME2 software identified 31 phyla, and at the genus level, sequences were classified into 346 different genera. As shown in Figure 1A, a total of 4,057 ASVs were identified collectively in the NAF, CF, and CAF groups. Among these, 971 ASVs were shared between the NAF and CF groups, 951 ASVs were shared between the NAF and CAF groups, and 832 ASVs were shared between the CAF and CF groups. Additionally, 596 ASVs were common to all three groups. Figure 1B illustrates the ASV distribution in the NAM, CM, and CAM groups, where a total of 5,201 ASVs were identified. The NAM and CM groups shared 1,148 ASVs, the NAM and CAM groups shared 1,487 ASVs, and the CAM and CM groups shared 1,149 ASVs. A total of 834 ASVs were common to all three groups.

### Effects of different starter feeds on the microbial diversity indices in the rumen of lambs

**Figure 2:**
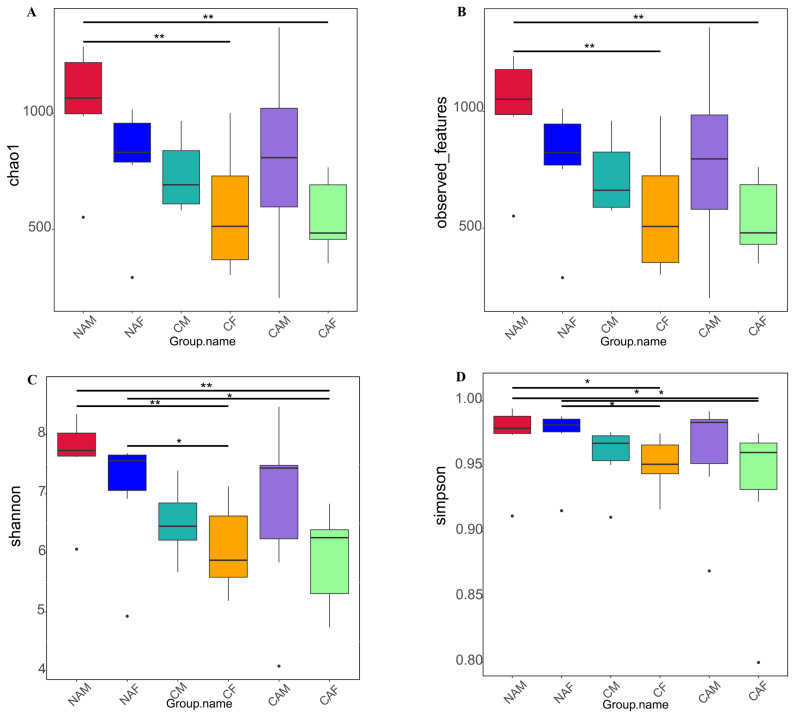
Rumen microbial diversity indices for lambs fed different starter feeds. Diversity indices include the Chao1 index (A), Observed_features index (B), Shannon index (C), and Simpson index (D). Statistical significance is indicated as follows: * p<0.05, ** p<0.01. NAM, standard pelleted starter feed without alfalfa, male lambs; NAF, standard pelleted starter feed without alfalfa, female lambs; CM, standard pelleted starter feed containing alfalfa, male lambs; CF, standard pelleted starter feed containing alfalfa, female lambs; CAM, starter feed with the same formulation as the CON group, but crumbled using a roller mill after pelleting, male lambs; CAF, starter feed with the same formulation as the CON group, but crumbled using a roller mill after pelleting, female lambs; F, female; M, male.

As shown in Figure 2, different starter feeds significantly influenced the alpha diversity indices of rumen microbes in lambs. In Figure 2A, the NAM group exhibited a significantly higher Chao1 index compared to the CF and CAF groups (p<0.01).

In Figure 2B, the Observed Features index for the NAM group was also significantly higher than that of the CF and CAF groups (p<0.01). In Figure 2C, the Shannon index of the NAM group was significantly greater than that of the CF and CAF groups (p<0.01). Additionally, the NAF group showed significantly higher values than the CF and CAF groups (p< 0.05). In Figure 2D, the Simpson index for the NAM group was significantly higher than that of the CF and CAF groups (p<0.05), while the NAF group also showed significantly higher values compared to the CF and CAF groups (p<0.05).

**Figure 3:**
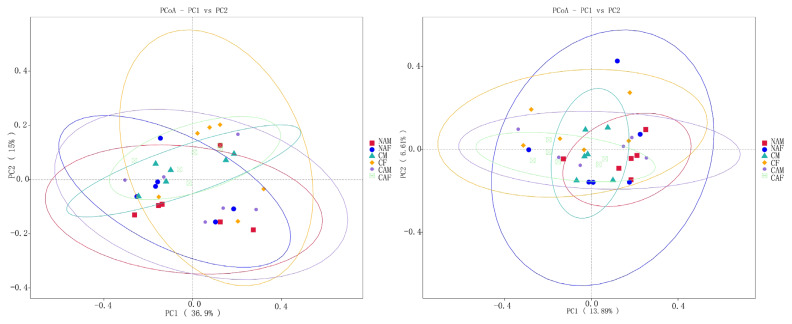
Beta diversity analysis of rumen microorganisms across different starter feed groups, visualized using principal coordinate analysis (PCoA). NAM, standard pelleted starter feed without alfalfa, male lambs; NAF, standard pelleted starter feed without alfalfa, female lambs; CM, standard pelleted starter feed containing alfalfa, male lambs; CF, standard pelleted starter feed containing alfalfa, female lambs; CAM, starter feed with the same formulation as the CON group, but crumbled using a roller mill after pelleting, male lambs; CAF, starter feed with the same formulation as the CON group, but crumbled using a roller mill after pelleting, female lambs; F, female; M, male.

As shown in Figure 3, PCoA analysis based on weighted UniFrac distances revealed that the first two principal coordinates explained 36.9% and 15% of the variance, respectively. In contrast, PCoA analysis using unweighted UniFrac distances indicated that the first two principal coordinates accounted for 13.89% and 6.61% of the variance. The lack of distinct clustering among different groups suggests that the various starter feeds did not significantly impact the beta diversity of rumen microbes in lambs.

### Effects of different starter feeds on the taxonomic abundance of rumen microbiota in lambs

**Figure 4:**
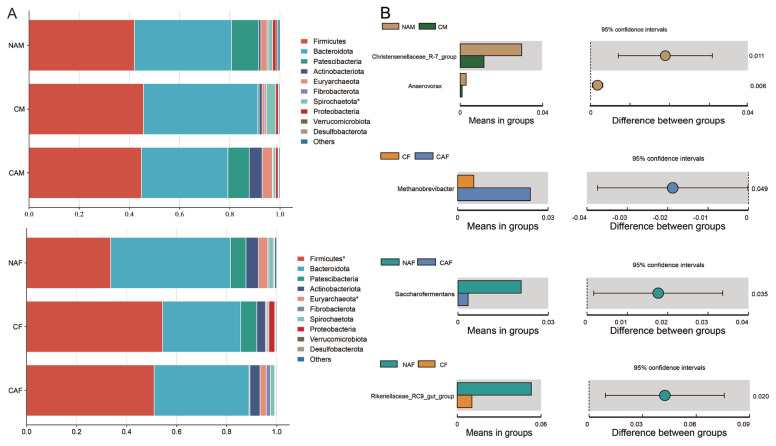
Relationship between rumen microbial composition and type of starter feed in lambs. (A) Differences in microbial composition among the groups at the phylum level. (B) Differences in microbial composition among the groups at the genus level. Statistical significance is indicated as follows: * p<0.05 (This notation applies only to Figure 4A). NAM, standard pelleted starter feed without alfalfa, male lambs; CM, standard pelleted starter feed containing alfalfa, male lambs; CAM, starter feed with the same formulation as the CON group, but crumbled using a roller mill after pelleting, male lambs; NAF, standard pelleted starter feed without alfalfa, female lambs; CF, standard pelleted starter feed containing alfalfa, female lambs; CAF, starter feed with the same formulation as the CON group, but crumbled using a roller mill after pelleting, female lambs; F, female; M, male.

As shown in Figure 4, the overall structure of the rumen microbial community was similar across all groups. At the phylum level, *Firmicutes* and *Bacteroidota* were the dominant phyla in all groups, with relative abundances exceeding 30%. At the genus level, the relative abundances of *Christensenellaceae R-7 group* and *Anaerovorax* were significantly higher in the NAM group compared to the CM group (p<0.05). The CAF group had a significantly higher abundance of *Methanobrevibacter* than the CF group (p<0.05). Additionally, the relative abundance of *Saccharofermentans* in the NAF group was significantly higher than in the CA group (p<0.05). *Rikenellaceae RC9 gut group* was more abundant in the NAF group compared to the CF group (p<0.05).

## DISCUSSION

The high-temperature conditioning process during feed pelleting is well-recognized for enhancing palatability and digestibility, which is why pelleted feed is a common choice for commercial lamb starter diets. However, the suitability of pelleted feed’s physical characteristics for lambs remains insufficiently explored. Previous research indicates that lambs fed pelleted starters exhibit lower feed intake and ADG compared to those fed textured starter feeds [9]. Similarly, studies in post-weaning lambs and calves show reduced ADG and DM intake with pelleted feeds compared to powdered or coarser feed forms [17]. These findings suggest that pelleted feed does not universally enhance growth performance in young ruminants.

The reduced intake of pelleted feed by lambs may be associated with its hardness. During the processing of pelleted feed, achieving a specific hardness level is crucial, as insufficient hardness can compromise pellet formation rates and durability [3]. This is particularly significant for starter feeds containing alfalfa, as the fiber content in alfalfa increases friction during the pelleting process. Furthermore, studies have shown that fiber’s high water-binding capacity reduces starch gelatinization by restricting swelling, heat transfer, and water ingress, which aligns with our experimental results showing alfalfa’s detrimental effect on pellet starch gelatinization [18]. While no current studies directly examine the effects of pellet hardness on lamb growth performance, higher hardness may negatively impact feed intake. In this study, removing alfalfa from the feed formulation resulted in a reduction in pellet hardness, though the difference was not statistically significant. Additionally, crumbling the pellets after pelleting substantially decreased their hardness and reduced their density, providing further support for the hypothesis.

This study notably found that feeding lambs crumbled pellets significantly increased their feed intake. Consistent with previous research, the feed intake of all lamb groups increased linearly with age, with particularly rapid growth observed after 21 days of age. In this experiment, the increase in feed intake for the crumbled pellet group after 21 days was significantly higher than that of the control group, with intake during the 21–35 day and 35–49 day periods being 2.30 times and 1.74 times greater, respectively. While limited research exists on the effects of starter feed type on lamb intake and growth, numerous studies on calves indicate that starter feed type significantly influences feed intake and growth [19]. For instance, calves tend to consume more when fed multiple small pellets or powdered feeds compared to standard pellets [20]. Though the use of crumbled pellets in ruminant diets has not been extensively studied, research in poultry has shown that chicks fed crumbled pellets achieved higher weights between 15 and 42 days of age compared to those fed whole pellets [21]. Similarly, studies in broilers during the growing phase reported that weight gain and feed-to-meat ratios were significantly improved with pellets and crumbled pellets compared to meal [22]. In this study, the substantial increase in feed intake for the crumbled pellet group (CA group) led to significantly higher ADG during the 35–49 day period compared to both the control and NA groups. Crumbled pellets maintain the advantages of pelleted feeds—such as high-temperature conditioning, reduced ingredient separation, and minimized feed waste—while also reducing hardness and particle size. These characteristics likely contributed to the significant increase in feed intake observed in lambs.

In this study, although the hardness of alfalfa-free pellets exhibited lower hardness (though not statistically significant) and significantly higher starch gelatinization compared to the control, lambs fed these pellets showed significantly reduced feed intake. This suggests that including alfalfa meal in starter feeds for young lambs benefits feed intake and development, consistent with findings from numerous studies [6]. For example, research on Tibetan sheep demonstrated that adding alfalfa to the diet significantly increased final BW [23]. Mechanistically, alfalfa stimulates proliferation of cellulolytic bacteria, promotes establishment of glycolytic microbiota, and increases short-chain fatty acid production [24], collectively modifying rumen fermentation patterns to enhance nutrient utilization efficiency. The effectiveness of roughage in supporting rumen development in young ruminants depends on several factors, including the type of roughage, fiber length, and supplementation methods [25]. Alfalfa’s high-quality fiber not only directly stimulates rumen development but also elevates blood β-hydroxybutyrate (BHBA) concentrations - a biomarker reflecting enhanced rumen wall metabolic activity [26]. These findings align with reports showing improved feed intake in calves fed straw-containing starters [27]. Therefore, simply removing alfalfa to address pelleting issues, such as increased friction or reduced starch gelatinization, is not effective. Instead, post-pelleting crumbling appears to be a more effective approach for optimizing pelleted feed performance.

This study further evaluated the impact of different starter feed types on the nutrient digestibility of lambs. Digestibility is influenced by various factors, including the physical form and composition of the feed. Research indicates that most starter feeds in lamb feeding practices are typically pelleted [28]. However, crumbled feeds or feeds with smaller particle sizes have demonstrated better nutrient digestibility compared to larger pellets [29]. The NDF and ADF digestibility in the NA group (standard pelleted feed without alfalfa) was significantly higher than in the CON group (standard pelleted feed with alfalfa). This difference may be attributed to the higher degree of starch gelatinization observed in the NA group. Previous research has shown that increasing the conditioning time during pelleting enhances starch gelatinization, subsequently improving the digestibility of DM and starch in calves [3]. Furthermore, the lower feed intake observed in the NA group may have contributed to the increased digestibility, as studies suggest that higher feed intake reduces retention time in the rumen, which can decrease nutrient digestibility. Interestingly, despite significantly higher feed intake in the CA group (crumbled pellets) compared to the CON group, the digestibility of NDF and ADF remained elevated in the CA group. This finding suggests that crumbled pellet feeds are easier for young lambs to digest and absorb. The physical structure of crumbled pellets appears to be more suitable for the underdeveloped digestive systems of young lambs. Additionally, increased feed intake may promote the physical growth and metabolic functions of the rumen, thereby enhancing fiber digestibility. Supporting this notion, related studies have shown that increased intake of solid feeds contributes to the growth in weight and volume of the rumen, enhancing its metabolic capacity [30].

This experiment further evaluated the effects of different types of starter feeds on the rumen fermentation function of lambs by analyzing VFA concentrations in rumen fluid. Among the VFAs, the type of starter feed significantly influenced only the propionate concentration, with no significant effects observed on total acid levels, other VFA concentrations, or the acetate-to-propionate ratio. While some studies suggest that concentrates generate higher propionate levels during fermentation [31], the NA group in this study exhibited lower propionate levels, likely due to reduced feed intake. The concentration of VFAs in rumen fluid reflects the balance between microbial fermentation and VFA absorption in the rumen. Other studies have similarly reported that young lambs supplemented with alfalfa exhibited lower propionate concentrations during the transitional period post-weaning [6]. The findings of this study indicate that variations in starter feed types and feed intake have minimal impact on the overall rumen fermentation pattern. Consistent with these results, other research emphasizes that the composition of the diet is the primary determinant of rumen fermentation type, whereas the physical form of the feed exerts minimal influence on fermentation parameters in sheep [32].

Numerous studies have demonstrated that the physical form of feed influences the rumen microbiota in lambs. For example, Liu et al reported that pelleted feed, compared to mash, affects phenotypic traits such as meat quality by modulating the rumen microbiota [33]. In light of this, we further investigated the effects of post-pelleting crumbling on the rumen microbiota of lambs. Our findings indicate that lamb sex influenced alpha diversity indices of the rumen microbiota. However, within the same sex, only diet composition—not post-pelleting crumbling—affected microbial diversity. Additionally, no significant differences in beta diversity were observed among the experimental groups. The observed differences in microbial composition are likely driven by variations in nutrient availability and fiber structure associated with different diet formulations. The inclusion or exclusion of certain feed ingredients can selectively promote or inhibit specific microbial populations, influencing overall diversity and fermentation dynamics [24]. In contrast, feed physical form, such as crumbling, does not substantially alter nutrient composition or fiber structure, which may explain its limited impact on microbial diversity. Similarly, Liu et al found no differences in the alpha diversity index of rumen microbiota between lambs fed pelleted versus non-pelleted feeds, aligning with the results of this study [33]. These findings suggest that diet composition plays a predominant role in modulating the rumen microbiota, while the physical form of feed, such as crumbling, has a minimal impact [34].

In terms of microbial composition, the dominant phyla across all groups were *Firmicutes* and *Bacteroidota*, with *Prevotella* being the dominant genus, consistent with previous studies [35]. This study found that pelleting or diet composition influenced the relative abundance of specific phyla and genera. Notably, the microbial groups that differed in male lambs were distinct from those in female lambs. This finding underscores the critical role of animal sex in shaping rumen microbiota and suggests that microbial responses to diet may vary between males and females. Consequently, the inclusion of both sexes in dietary and microbiome studies is vital to obtain a comprehensive understanding of microbial dynamics and dietary impacts, ensuring the broad applicability of findings across populations. Compared to physical feed form, alfalfa inclusion had a more significant influence on a broader range of microbial genera, including the *Christensenellaceae* R-7 group, *Anaerovorax*, *Saccharofermentans*, and *Rikenellaceae* RC9 gut group, all of which exhibited higher relative abundances in the NA. These results align with other studies, which have similarly reported increased abundances of the *Christensenellaceae* R-7 group [36], *Saccharofermentans*, and *Rikenellaceae* RC9 gut group [37] in high-concentrate diets. This suggests that these genera are sensitive to the forage-to-concentrate ratio, with the chemical composition of high-concentrate diets creating specific niches that favor their proliferation.

Our study also found that post-pelleting crumbling increased the abundance of *Methanobrevibacter*. Numerous studies examining the relationship between feed efficiency and rumen microbiota in ruminants have reported an association between *Methanobrevibacter* abundance and feed conversion efficiency, with higher *Methanobrevibacter* abundance often observed in low feed-efficiency groups [38]. A consistent observation across these studies is that increased feed intake correlates with greater *Methanobrevibacter* abundance. In this study, the post-pelleting crumbling group exhibited a notable increase in feed intake accompanied by a significant rise in *Methanobrevibacter* abundance, aligning with these findings. Similarly, research on rabbits demonstrated that smaller pellet sizes enhanced both feed intake and cecum *Methanobrevibacter* abundance [39], further supporting our results. These findings underscore the role of feed intake as a key factor influencing *Methanobrevibacter* abundance in the rumen. While the physical form of feed, particularly post-pelleting crumbling, did not significantly affect microbial diversity indices, it did influence the abundance of specific microbial groups. This study suggests that the physical form of feed can indirectly affect microbial composition by altering intake behavior.

These findings highlight the practical value of post-pelleting crumbling for lamb starter feeds in commercial production systems. By combining alfalfa retention with mechanical crumbling, producers can effectively address the common trade-off between pellet durability and palatability for young lambs. The improved feed intake and growth performance observed in this study suggest that this approach better meets the physiological needs of developing lambs compared to conventional pelleting or alfalfa-free formulations. These results highlight the importance of considering both physical structure of feed and its dietary composition to optimize intake and rumen development, providing a practical solution for modern lamb production systems.

## CONCLUSION

This study demonstrates that crumbling pelleted feed significantly improves feed intake and growth performance in young lambs by reducing pellet hardness and enhancing fiber digestibility. While removing alfalfa from the starter feed increased pellet starch gelatinization and lamb fiber digestibility, it also resulted in reduced feed intake. Feed composition had a greater impact on rumen microbial diversity than the feed form. Crumbling specifically increased the abundance of *Methanobrevibacter*. Overall, these findings suggest that crumbled starter feed is a practical and effective strategy for improving feed intake, nutrient digestibility, and growth performance in lambs.

## Figures and Tables

**Table 1:** Dietary formulation and nutrient levels (air-dry basis)

Ingredients (%)	Treatment group	

	CON and CA	NA
Alfalfa	7.90	-
Corn	37.30	40.30
Extruded soybeans	2.50	2.50
Whey powder	1.50	1.50
Corn bran	6.35	9.25
Soybean meal	18.25	19.25
Cottonseed meal	5.00	5.00
DDGS	12.00	13.00
Bio-fermented feed	2.50	2.50
Limestone	1.70	1.70
Premix	5.00	5.00
Total	100.00	100.00
Chemical composition	Content	
DM (%)	93.55	93.01
DE (MJ·kg^−1^)	12.68	13.00
CP (%)	22.38	22.58
NDF (%)	16.27	14.00
ADF (%)	7.37	6.35
Ca (%)	1.38	1.20
P (%)	0.38	0.50

The premix composition per kilogram of diet is as follows: 25 mg Fe as FeSO_4_·H_2_O; 40 mg Zn as ZnSO_4_·H_2_O; 8 mg Cu as CuSO_4_·5H_2_O; 40 mg Mn as MnSO_4_·H_2_O; 0.3 mg I as KI; 0.2 mg Se as Na_2_SeO_3_; 0.1 mg Co as CoCl_2_; 940 IU vitamin A; 111 IU vitamin D; 20 IU vitamin E, and; 0.02 mg vitamin B_12_. DM, CP, NDF, ADF, Ca, and P were measured values, while DE was calculated in reference to [40].

CA, crumbled alfalfa; NA, non-alfalfa; DM, dry matter; DE, digestible energy; CP, crude protein; NDF, neutral detergent fiber; ADF, acid detergent fiber.

**Table 2:** Physical properties and starch gelatinization of starter feeds with different forms

Treatment	Hardness (ѱ N)	Density (ρ g/cm^3^)	Starch gelatinization (%)
CON	230.94^a^	0.90	22.36^b^
NA	205.11^a^	0.92	33.76^a^
CA	27.34^b^	0.82	23.65^b^
SEM	10.473	0.073	0.020
p-value	<0.001	0.397	0.002

a,b Values within a column with different superscripts differ significantly at p<0.05.

CON, fed a standard pelleted starter feed containing alfalfa; NA, fed a standard pelleted starter feed without alfalfa; CA, fed a starter feed with the same formulation as the CON group but crumbled using a roller mill after pelleting; SEM, standard error of the mean.

**Table 3:** Effects of different starter feed form and sex on lamb growth performance

Items	Groups						Main effects					SEM	p-value		
	
	F			M			CON	NA	CA	F	M		Feed	Sex	F×S
	
	CON	NA	CA	CON	NA	CA									
BW (kg)															
BW7 (kg)	3.83	3.81	3.79	4.20	4.10	4.52	4.02	3.95	4.15	3.81	4.27	0.090	0.636	0.008	0.564
BW21 (kg)	5.49	5.45	5.31	6.25	6.15	6.00	5.87	5.80	5.65	5.42	6.13	0.120	0.770	0.005	0.992
BW35 (kg)	7.02	6.76	6.88	8.03	7.65	8.02	7.52	7.20	7.45	6.89^b^	7.90^a^	0.170	0.733	0.004	0.959
BW49 (kg)	8.48	7.93	9.43	9.43	8.96	10.06	8.96	8.44	9.74	8.61	9.48	0.230	0.080	0.061	0.934
ADG (kg)															
ADG7–21 (kg)	0.12	0.12	0.11	0.15	0.15	0.11	0.13^a^	0.13^a^	0.11^b^	0.11	0.13	0.000	0.030	0.039	0.258
ADG21–35 (kg)	0.11	0.09	0.11	0.13	0.11	0.14	0.12	0.10	0.13	0.10	0.13	0.010	0.142	0.063	0.798
ADG35–49 (kg)	0.10	0.08	0.18	0.10	0.09	0.15	0.10^b^	0.09^b^	0.16^a^	0.12	0.11	0.010	0.000	0.463	0.398
ADG7–49 (kg)	0.11	0.10	0.13	0.12	0.12	0.13	0.12^ab^	0.11^b^	0.13^a^	0.11	0.12	0.000	0.049	0.254	0.609

a,b Values within a row with different superscripts differ significantly at p<0.05.

F, female; M, male; CON, fed a standard pelleted starter feed containing alfalfa; NA, fed a standard pelleted starter feed without alfalfa; CA, fed a starter feed with the same formulation as the CON group but crumbled using a roller mill after pelleting; BW, body weight; ADG, average daily gain.

**Table 4:** Effects of different starter feed form on starter intake

Items	Main effects			SEM	p-value

	CON	NA	CA		
FI 7–21 (g)	6.66^b^	7.32^b^	9.44^a^	0.191	<0.001
FI 21–35 (g)	25.28^b^	17.25^c^	57.99^a^	1.823	<0.001
FI 35–49 (g)	92.15^b^	50.92^c^	159.85^a^	5.066	<0.001
FI 7–49 (g)	41.36^b^	25.16^c^	75.76^a^	2.281	<0.001

a–c Values within a row with different superscripts differ significantly at p<0.05.

CON, fed a standard pelleted starter feed containing alfalfa; NA, fed a standard pelleted starter feed without alfalfa; CA, fed a starter feed with the same formulation as the CON group but crumbled using a roller mill after pelleting; SEM, standard error of the mean.

**Table 5:** Effects of different starter feed form and sex on nutrient digestibility

Items	Groups						Main Effects					SEM	p-value		
	
	F			M			CON	NA	CA	F	M		Feed	Sex	F×S
	
	CON	NA	CA	CON	NA	CA									
CP (%)	0.62	0.62	0.85	0.82	0.78	0.84	0.72^b^	0.70^b^	0.85^a^	0.70^b^	0.81^a^	0.024	0.060	0.027	0.216
NDF (%)	0.43^b^	0.67^a^	0.67^a^	0.60^a^	0.67^a^	0.64^a^	0.51^b^	0.67^a^	0.66^a^	0.59	0.64	0.014	0.001	0.130	0.028
ADF (%)	0.36^b^	0.54^a^	0.59^a^	0.52^ab^	0.63^a^	0.45^ab^	0.44^b^	0.59^a^	0.52^a^	0.50	0.53	0.014	0.003	0.215	0.001
DM (%)	0.80	0.79	0.76	0.73	0.76	0.80	0.77	0.78	0.78	0.78	0.76	0.018	0.956	0.681	0.382
OM (%)	0.81	0.80	0.78	0.74	0.78	0.82	0.78	0.79	0.80	0.80	0.78	0.017	0.900	0.534	0.423

a,b Values within a row with different superscripts differ significantly at p<0.05.

CON, fed a standard pelleted starter feed containing alfalfa; NA, fed a standard pelleted starter feed without alfalfa; CA, fed a starter feed with the same formulation as the CON group but crumbled using a roller mill after pelleting; F, female; M, male; CP, crude protein; NDF, neutral detergent fiber; ADF, acid detergent fiber; DM, dry matter; OM, organic matter.

**Table 6:** Effects of different starter feed form and sex on rumen fermentation of lambs

Items	Groups						Main effects					SEM	p-value		
	
	F			M			CON	NA	CA	F	M		Feed	Sex	F×S
	
	CON	NA	CA	CON	NA	CA									
Acetic acid (mmol/L)	45.73	44.17	44.15	48.43	38.40	36.83	47.08	41.28	40.49	44.68	41.22	2.368	0.473	0.471	0.653
Propionic acid (mmol/L)	14.64	12.38	15.43	15.48	10.23	9.72	15.06^a^	11.31^b^	12.57^ab^	14.15	11.81	0.518	0.019	0.031	0.050
Isobutyric acid (mmol/L)	1.78	1.48	1.90	1.42	1.15	1.07	1.60	1.31	1.48	1.72	1.21	0.098	0.497	0.014	0.520
Butyric acid (mmol/L)	5.06	4.42	6.11	6.44	3.48	3.99	5.75	3.95	5.05	5.20	4.64	0.365	0.146	0.448	0.155
Isovaleric acid (mmol/L)	0.84	0.86	1.22	0.93	0.52	0.72	0.88	0.69	0.97	0.97	0.72	0.075	0.312	0.110	0.273
Valeric acid (mmol/L)	0.74	0.64	0.81	0.76	0.57	0.60	0.75^a^	0.61^b^	0.71^a^	0.73	0.65	0.025	0.080	0.107	0.187
Total acid (mmol/L)	71.67	66.83	72.50	76.34	57.24	55.81	74.00	62.04	64.16	70.34	63.13	3.168	0.273	0.264	0.386
Acetate/propionic acid	3.13	3.48	2.88	3.09	3.75	3.68	3.11	3.61	3.28	3.16	3.51	0.107	0.171	0.120	0.284

a,b Values within a row with different superscripts differ significantly at p<0.05.

F, female; M, male; CON, fed a standard pelleted starter feed containing alfalfa; NA, fed a standard pelleted starter feed without alfalfa; CA, fed a starter feed with the same formulation as the CON group but crumbled using a roller mill after pelleting.
